# *HOXB7* mRNA is overexpressed in pancreatic ductal adenocarcinomas and its knockdown induces cell cycle arrest and apoptosis

**DOI:** 10.1186/1471-2407-13-451

**Published:** 2013-10-02

**Authors:** Thais Chile, Maria Angela Henriques Zanella Fortes, Maria Lúcia Cardillo Corrêa-Giannella, Helena Paula Brentani, Durvanei Augusto Maria, Renato David Puga, Vanessa de Jesus R de Paula, Marcia Saldanha Kubrusly, Estela Maria Novak, Telésforo Bacchella, Ricardo Rodrigues Giorgi

**Affiliations:** 1 Laboratory for Cellular and Molecular Endocrinology (LIM-25), University of São Paulo Medical School, Av. Dr. Arnaldo, 455 # 4305, São Paulo, SP, 01246-903, Brazil; 2 Department of Gastroenterology (LIM-07 and 37), University of São Paulo School of Medicine, Sao Paulo, SP, Brazil; 3 Institute of Psychiatry - University of Sao Paulo, Medical School (FMUSP), São Paulo, SP, Brazil; 4 Laboratory of Biochemistry and Biophysics, Butantan Institute, Av. Vital Brazil, 1500, São Paulo, 05503-900, Brazil; 5 CIPE - AC Camargo Hospital, São Paulo, SP, Brazil; 6 Institute of Psychiatry - University of Sao Paulo, Medical School (FMUSP), São Paulo, SP, Brazil; 7 Laboratory Molecular Biology- Fundação Pró-Sangue Hemocentro of São Paulo, São Paulo, SP, Brazil; 8 Pediatric Clinical Laboratory (LIM 36), University of São Paulo, Medical School (FMUSP), São Paulo, SP, Brazil

**Keywords:** Pancreatic ductal adenocarcinoma, Homeobox, *HOXB7*, siRNA, Gene expression

## Abstract

**Background:**

Human homeobox genes encode nuclear proteins that act as transcription factors involved in the control of differentiation and proliferation. Currently, the role of these genes in development and tumor progression has been extensively studied. Recently, increased expression of *HOXB7* homeobox gene (*HOXB7*) in pancreatic ductal adenocarcinomas (PDAC) was shown to correlate with an invasive phenotype, lymph node metastasis and worse survival outcomes, but no influence on cell proliferation or viability was detected. In the present study, the effects arising from the knockdown of *HOXB7* in PDAC cell lines was investigated.

**Methods:**

Real time quantitative PCR (qRT-PCR) (Taqman) was employed to assess *HOXB7* mRNA expression in 29 PDAC, 6 metastatic tissues, 24 peritumoral tissues and two PDAC cell lines. siRNA was used to knockdown *HOXB7* mRNA in the cell lines and its consequences on apoptosis rate and cell proliferation were measured by flow cytometry and MTT assay respectively.

**Results:**

Overexpression of *HOXB7* mRNA was observed in the tumoral tissues and in the cell lines MIA PaCa-2 and Capan-1. *HOXB7* knockdown elicited (1) an increase in the expression of the pro-apoptotic proteins BAX and BAD in both cell lines; (2) a decrease in the expression of the anti-apoptotic protein BCL-2 and in cyclin D1 and an increase in the number of apoptotic cells in the MIA PaCa-2 cell line; (3) accumulation of cell in sub-G1 phase in both cell lines; (4) the modulation of several biological processes, especially in MIA PaCa-2, such as proteasomal ubiquitin-dependent catabolic process and cell cycle.

**Conclusion:**

The present study confirms the overexpression of *HOXB7* mRNA expression in PDAC and demonstrates that decreasing its protein level by siRNA could significantly increase apoptosis and modulate several biological processes. *HOXB7* might be a promising target for future therapies.

## Background

PDAC is one of the most frequent causes of cancer-related death worldwide. It is an aggressive neoplasia whose early diagnosis and treatment are challenging, making it a leading cause of death by cancer [1]. Most patients are diagnosed at an advanced stage and only a few of these patients are suitable candidates for curative surgery [2,3]. Homeobox-containing genes encode DNA-binding proteins that regulate gene expression and control various aspects of morphogenesis and cell differentiation [4]. In humans, *HOX* genes are represented by 39 members classified in four groups (*HOX-A*, *HOX-B*, *HOX-C* and *HOX-D*) located on chromosomes 7p, 17q, 12q and 2q, respectively. Aberrant expression of homeobox genes have been shown in different tumour types [5-9], including leukemias [10,11], ovarian carcinoma [12], and breast cancer [13]. The gene expression of *HOXB5*, *HOXB6*, *HOXC8* and *HOXD13* have already been characterized in pancreatic cancer [14]. *HOXB7* has an important role in various tumors. In melanomas, overexpression of *HOXB7* constitutively activates basic fibroblast growth factor (bFGF), favoring uncontrolled cell proliferation [15]. In a breast cancer cell line (SkBr3), transduction of *HOXB7* gene induces bFGF expression, increases growth rate and ability of cells to form colonies in semisolid medium [16]. In addition to *bFGF*, *HOXB7* can also induce the expression of other genes, especially those related to angiogenesis and tumor invasion including vascular endothelial growth factor (*VEGF*), interleukin-8, angiopoietin-2, and metalloproteases 2 and 9 [17]. Increased expression of *HOXB7* was also described in oral squamous cell carcinoma, where it induces cell proliferation and has been shown to be associated with poor prognosis [18]. In colorectal cancer, the protein encoded by *HOXB7* was considered as a prognostic factor and mediator of tumor development and progression [19]. Recently *HOXB7* status was investigated in a large cohort of PDAC, the authors observed overexpression of *HOXB7* and its correlation with invasive phenotype, lymph node metastasis and worse survival outcomes, but no influence on cell proliferation or viability was detected [20]. The aim of this study was to further investigate *HOXB7* expression in PDAC and metastatic tissues in comparison to normal pancreatic and peritumoral tissues as well as to evaluate the effects of *HOXB7* knockdown in pancreatic cancer cell lines, addressing cell proliferation, apoptosis and gene expression profile.

## Methods

### Patients and tumor characterization

Tissue collection was carried out in compliance with The Ethical Committee of Hospital das Clínicas (Faculdade de Medicina da Universidade de São Paulo) and in accordance to The Declaration of Helsinki, with informed and free consent obtained from each subject. The following tissue samples were obtained from patients diagnosed with PDAC: tumoral (n=29), disease-free tissues (located distant from the tumor site, n=24) and metastatic tissues (liver metastasis, n=6). Ten normal pancreatic tissue samples obtained within 8 hours post-mortem from subjects without pancreatic diseases were used as control. The diagnosis was established by clinical, biochemical, and radiological findings and supported by the anatomopathological analysis of tumor samples.

During surgical procedure, tumor fragments were collected in sterile containers with 1 mL of RNA*later®* (Ambion, Inc., Austin, TX, USA) and stored at 4°C. All tumoral, disease-free and metastatic samples were resected by a experienced surgeon.

### RNA and DNA extraction

The material collected in RNA*later®* (Ambion) was fragmented in a tissue pulverizer (Mikro-Dismembrator U, B. Braun Biotech International, Melsungen, Hesse, Germany). Total RNA was extracted from approximately 100 mg tissue after homogenization, using with RNeasy Plus Mini Kit (Qiagen, Duesseldorf, North Rhine-Westphalia, Germany) according to manufacturer’s guidelines. DNA was extracted using the DNeasy kit (Qiagen) according to the manufacturer’s instructions.

Both were measured spectrophotometrically being adopted values of optical density 260/280 nm and 260/230 nm between 1.8 and 2.0. A integrity of RNA was checked by visual inspection of the 18S e 28S ribosomal RNA bands in 1% agarose gel, while DNA integrity was verified by the presence of a single band in agarose gel 2%.

### Validation of endogenous reference gene

**Figure 1:**
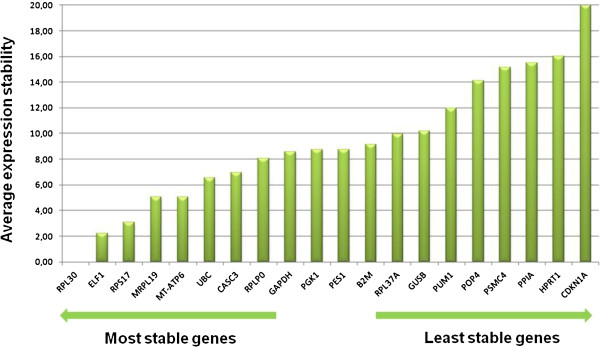
RPL30 gene showed the least variation of expression among all tested housekeeping genes in samples from normal pancreatic tissue and pancreatic ductal adenocarcinomas.

In order to determine the most stable gene and to normalize the target gene in pancreatic tissues, we studied the expression of 32 commonly used reference genes. The expression of candidate genes was evaluated with the TaqMan Express Endogenous Control Plate, according to the manufacturer’s protocol (Applied Biosystems, Foster City, CA, USA). The genes are performed in triplicate in these arrays and are constitutively expressed at moderate abundance across most test samples. cDNA was prepared from ten samples of normal pancreatic tissue and ten samples of PDAC using SuperScript™ III Reverse Transcriptase (Invitrogen Corporation, Carlsbad, CA, USA). Gene expression was measured by quantitative real time qRT-PCR and expression stability was analyzed with geNorm [21] and NormFinder [22]. Based on the results of this analysis, *RPL30* was proposed as the most appropriate control gene (Figure 1).

### Quantitative real-time polymerase chain reaction after reverse transcription (qRT-PCR)

Complementar DNA (cDNA) was synthesized from total RNA extracted from each cell line and tissue samples. Briefly, first-strand cDNA synthesis used 1 μg of total RNA, 1 μL of oligo(dT) primers (0.5 μg/μL), 1 μL of a solution of all four deoxyribonucleoside triphosphates (each at 10 mM), and 10× SuperScript™ III Reverse Transcriptase (Invitrogen Corporation). For TaqMan-based qRT-PCR, 50 ng of cDNA was added to 10 μL of 2× Taqman Universal PCR Master Mix (Applied Biosystems) and 1 μL of 20× *HOXB7* primers and the probe set (Applied Biosystems). The one-step RT-PCR was performed using a StepOne Plus (AB Applied Biosystems) for an initial 2 minutes incubation at 50°C, 10 minutes incubation at 95°C followed by 40 cycles of PCR 95°C for 15 seconds and 60°C for 1 minute. Data values (Cycle Threshold [Ct] values) were extracted from each assay with the SDS v2.0 software tool (Applied Biosystems).

The number of specific (*HOXB7*) transcripts in tumor samples was normalized to housekeeping gene *RPL30* mRNA in three independent experiments. Glyceraldehydes-3-phosphate dehydrogenase (*GAPDH)* was used as denominators of gene expression in cell lines. Gene expression levels were analyzed by the comparative Ct method (ΔΔCt) [23].

### Copy number analysis of HOXB7 by real-time quantitative PCR (qPCR)

*HOXB7* amplification was assessed by qPCR using Platinum® SYBR® Green qPCR SuperMix-UDG (Invitrogen Corporation). Beta-2-microglobulin (*β2M*) was used as reference gene for the evaluation of *HOXB7* copy number.

Genomic DNA (100 ng/μL) from each tissue sample was conducted on a Applied Biosystems StepOne Plus (Applied Biosystems, Foster City, CA, USA) using the following primers for genomic sequences of *HOXB7* (sense: 5′- CGA TGC AGG GCT TGT ACC -3′; anti-sense: 5′- AGG CGC CTT CAG GGT AAT -3′) and *β2M* (sense: 5′- CGT GTG AAC CAT GTG ACT TTG -3′; anti-sense: 5′- GAA TTC ATC CAA TCC AAA TGC -3′). The reaction was incubated for 5 minutes at 94°C, followed by 40 cycles of 30 seconds at 94°C, 30 seconds at 55°C, and 90 seconds at 72°C, with a final extension of 72°C for 7 minutes. All samples were run in duplicate and positive *HOXB7* gene amplification was defined as a copy number of > 3 [24].

### Cell culture

Human pancreatic cancer cell line MIA PaCa-2 was obtained from American Type Culture Collection (ATCC® Number: CRL-1420™, Manassas, VA, USA). The cells were maintained routinely in Roswell Park Memorial Institute (RPMI) 1640 medium (Invitrogen Corporation, Carlsbad, CA, USA) supplemented with 10% fetal bovine serum (FBS, Invitrogen Corporation), 100 U/mL penicillin G (Invitrogen Corporation), and 0.1 mg/mL streptomycin sulfate (Invitrogen Corporation) at 37°C in a humidified, 5% CO_2_, 95% air atmosphere. Capan-1 cell line established from a hepatic metastasis of a PDAC was also obtained from ATCC (Number: HTB-79™). The cells were grown in IMDM medium (Invitrogen Corporation) supplemented with 20% FBS (Invitrogen Corporation).

### RNAi knockdown (siRNA) and transfection

The human pancreatic cancer cell lines were cultured as described. siRNA and transfections were performed following the manufacturer’s protocols of the TriFECTa Dicer- Substrate RNAi kit (IDT, Coralville, IA, USA) and Lipofectamine RNAi Max Reagent (Invitrogen Corporation). 10^5^ cells were plated in 6-well in RPMI medium one day prior to transfection. Cells were transfected with a nonspecific scrambled siRNA and with a *HOXB7*-specific siRNA at a final concentration of 10 nM. The mRNA content was measured 48 hours after transfection. All transfections were minimally performed in duplicate. *HOXB7* depletion and RT-qPCR were performed as described above. Each experiment was repeated at least twice.

### Western blotting

After 48 h electroporation with siRNAs, cells were homogenized in RIPA buffer (Cell Signaling Technology, Danvers, MA, USA) with protease inhibitors (Complete, Mini, EDTA-free Protease Inhibitor Cocktail Tablet, Roche Applied Science, Penzberg, Upper Bavaria, Germany). The homogenate was centrifuged at 16,700 g for 30 minutes at 4°C. Protein concentration was measured using Lowry method [25].

Thirty micrograms of total protein was separated on a 14% sodium dodecyl sulfate polyacrylamide gel followed by transfering to an Immobilon-P membrane (Merck Millipore, Billerica, MA, USA). Membranes were incubated for 18 hours in 5% skim milk phosphate buffer saline (PBS) with mouse monoclonal antibody *HOXB7* (1:50, ab51237, Abcam Inc, Cambridge, MA, USA) followed by incubation with secondary antibody (1:400, RPN1001, GE Healthcare, Little Chalfont, Buckinghamshire, UK) and labeled with horseradish peroxidase (1:3000, GE Healthcare). Rabbit anti-beta actin antibody (1:1000, ab8227, Abcam Inc, Cambridge, MA, USA) was used as internal control. Photographic film was exposed to the membrane in a dark room.

### MTT cell proliferation assay

Cell proliferation was evaluated after 24 hours, 48 hours and 72 hours after transfection with siRNA-*HOXB7* using a specific colorimetric assay. In particular, cells were exposed to *HOXB7* siRNA and then stained with 3-(4,5-dimethylthiazol-2-yl) – 2,5-diphenyltetrazolium bromide (MTT, Sigma-Aldrich, St Louis, MO, USA). The absorbance was measured by ELx 808 Ultra Microplate Reader (Bio-Tek Instruments, Inc, Winooski, VT, USA) at a wavelength of 570 nm.

### Flow cytometry – markers, cell cycle distribution, and apoptosis analysis

Forty-eight hours after transfection, the human pancreatic cells lines were trypsinized and inactivated with FBS, centrifuged at 1,500 rpm for 10 min, and the supernatant was discarded. The pellet was resuspended in 5 mL of PBS at a concentration of 10^6^ cells/mL. To analyze intracytoplasmic and nuclear markers, cells were permeabilized with 5 μL of 0.1% Triton X-100 for 30 min before the addition of specific primary antibodies. The following markers were used to determine cell death pathways: Bax (Ab5714, Abcam Inc), Bad Ab32445, Abcam Inc), and Bcl-2 (Ab692, Abcam Inc). Antibodies for cyclin D1 (sc8396, Santa Cruz Biotechnology Inc, Santa Cruz, CA, USA) were used to determine the proliferation index. The samples were analyzed in a flow cytometer (FACSCalibur, BD, Franklin Lakes, NJ, USA), and expression of cell proliferation and cell death markers were compared with parental control cells.

Detection of the markers was followed by analysis of the cell cycle phases. In this step, the trypsinized cells were treated with 70% ice-cold ethanol containing 100 μg/mL RNase. They were then washed and incubated in PBS at 37°C for 45 minutes. The labeling was performed in a solution containing propidium iodide (PI) at a concentration of 1.8 mg/mL to assess the integrity and quantity of DNA in the cell cycle phases.

Evaluation of apoptosis was carried out using Annexin V FITC Apoptosis Detection kit I (BD) according to the manufacturer’s instructions. Cells were centrifuged and the cell pellet was suspended with binding buffer (100 μL) and then incubated with Annexin V-FITC (2 μL) and PI (2 μL) for 15 minutes, at room temperature in the dark. After incubation, 400 μL of binding buffer was added and cells were analyzed in a FACScalibur (BD) using CellQuest software for determining the percentage of apoptotic cells. A minimum of 10,000 events was acquired for each sample [26].

### Microarray analysis after knockdown of HOXB7

Total RNA derived from the inhibition of gene transcript *HOXB7* as well as from parental cells were quantified in Bioanalyzer (Agilent, Santa Clara, CA, USA). This procedure was performed in duplicate for all cell lines, which were sorted into treated and untreated with siRNA. Each reaction was prepared from 200 ng of total RNA in a volume of 1,5 μL. The guidelines of the protocol One-Color Microarray-Based Gene Expression (Agilent, Santa Clara, CA, USA) were followed with the use of Agilent Low Input Quick Amp Labeling Kit. Hybridized slides (Human 4x44K Microarray) were washed as recommended and scanned using the High-Resolution Microarry Scanner (Agilent, Santa Clara, CA, USA). Data were extracted with Agilent Technologies Feature Extraction Software version 9.5.3.

### Validation of microarray assay

Validation of microarray was performed from the analysis of E2F and RB1 mRNA expression in Mia PaCa-2 cell line by RT-qPCR. The experiment was performed as described previously.

### Statistical analysis

For analysis of *HOXB7* expression and amplification statistical tests were two-tailed, with statistical significance fixed at 0.05. Continuous variables were analyzed using Kruskal-Wallis and Mann–Whitney U nonparametric tests. Values were expressed as median, minimum and maximum values. Data were analyzed using JMP Software version 8 (SAS Institute Inc, Cary, NC, USA).

Statistical analysis of MTT and flow cytometry was performed by analysis of variance (ANOVA) with the multiple comparison test of Tukey-Kramer. Values were expressed as mean ± standard deviation, considering as significant p values < 0.05.

Analysis of data obtained from the microarray experiment was performed using the self-HT [27]. The self-self experiments were performed with duplicates untreated labeled with Cy3 (untreated lineage x untreated lineage), assuming, then, that the variability of signal in microarray experiments is dependent of the intensity and any difference in hybridization is product of experimental artifact. From the self-self, a credibility interval of 99% was established to differentiate changes in expression of technique artifact, resulting therefore in determining intensity-dependent cutoffs, which were used in the experiments non-self-self (treated lineage x untreated lineage). On the platform array, the same gene is shown more than once by different probes, therefore, three criteria have been defined for identifying genes differentially expressed: (1) each gene was represented by at least two probes; (2) more than 50% of the probes representing one particular gene presented signal after expression quality analysis; (3) there was 100% agreement between the probe signal (up regulation or down regulation). Microarray data are available through the Minimum Information About a Microarray Experiment (MIAME, accession number GSE46393).

Two lists of differentially expressed genes were generated for each cell line, one containing the upregulated genes and other presenting downregulated genes common to the experimental duplicates. Each list was annotated in categories of biological processes according to the Gene Ontology database and the analysis was performed in WebGestalt [28]. The results were seen in directed acyclic graphs to maintain the relationship between categories enriched.

Hypergeometric test was used to evaluate the categorical enrichment and as multiple categories were tested simultaneously, *p* values were adjusted according to the adjustment method of multiple test proposed by Benjamini and Hochberg [29]. The significance for enrichment analysis was fixed at 0.01. Furthermore, a minimum number of two genes were established as the cutoff required.

## Results

### HOXB7 mRNA expression in pancreatic tissue samples and cell lines

**Figure 2:**
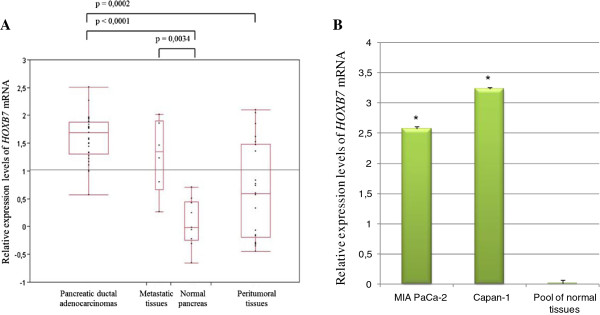
**Relative expression levels of HOXB7 mRNA.** Panel **A** depicts normalized expression values in pancreatic tissues. The horizontal line within the box plot represents the median value, the box plot limits refer to 25th to 75th percentiles, and the box plot bars include the 10th to 90th percentiles. Panel **B** indicates normalized expression values in pancreatic cell lines (MIA PaCa-2 and Capan-1) and pool of normal tissues. The experiments were carried in triplicate and are represented as mean ± standard deviation *p= 0,01.

*HOXB7* mRNA expression was analyzed in 29 pancreatic ductal adenocarcinoma samples, 24 peritumoral tissue samples, 6 metastatic tissues samples, and 10 normal pancreatic tissue samples. A higher content of *HOXB7* mRNA was observed in tumoral and in metastatic tissues in comparison to normal pancreas (control) (Figure 2A). *HOXB7* mRNA overexpression was also observed in MIA PaCa-2 and Capan-1 cell lines (Figure 2B).

**Figure 3:**
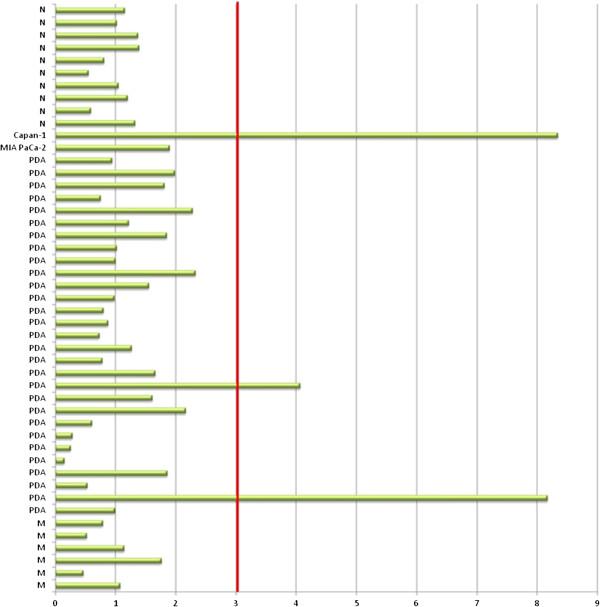
**HOXB7 gene copy number detected by quantitative PCR in pancreatic tissues and two cell lines.** Positive amplification was defined as ≥ 3 copies. N- normal pancreas; PDA- pancreatic ductal adenocarcinoma; M- metastatic tissue.

The number of copies of *HOXB7* was determined in all tissue samples and in both cell lines with the purpose of investigate the possibility of genomic amplification. As shown in Figure 3, only two tumoral samples and the Capan-1 cell line presented more than three copies of *HOXB7* gene.

### HOXB7 silencing evaluation

**Figure 4:**
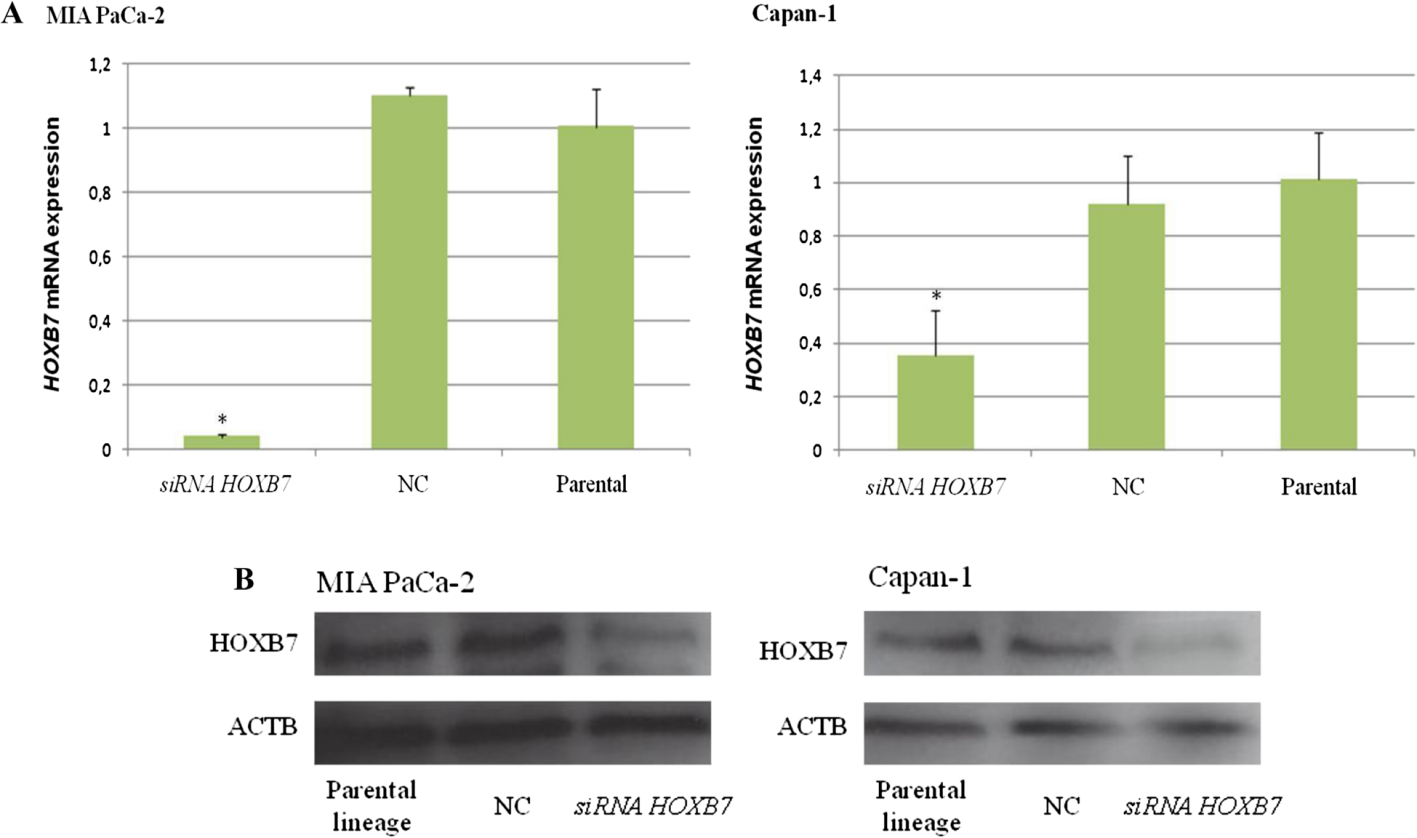
**HOXB7 gene expression 48 hours after transfection of siRNA.** Panel **A** depicts relative expression levels of HOXB7 mRNA in MIA PaCa-2 (*p=0.0270) and Capan-1 (*p=0.0003) cells lines; the experiments were carried in triplicate and are represented as mean ± standard deviation. Panel **B** depicts HOXB7 protein expression; beta-actin was used as internal control. NC- negative control.

The two human pancreatic cell lines MIA PaCa-2 and Capan-1 were transiently transfected with two siRNA duplexes targeting different encoding regions of human *HOXB7* mRNA, named as siRNA1 and siRNA2 or a nonspecific scrambled siRNA control. After 48 hours, the *HOXB7* mRNA and protein levels were quantified by real time RT-PCR and western blot, respectively. *HOXB7* siRNA significantly silenced the content of *HOXB7* mRNA in both pancreatic cell lines while the scrambled siRNA had no effect (Figure 4A). Approximately 96% and 65% of *HOXB7* mRNA were silenced in MIA PaCa-2 and Capan-1 cells, respectively. Western Blotting analysis also demonstrated that *HOXB7* siRNAs decreased proteins level in both cell lines (Figure 4B).

### MTT assay

**Figure 5:**
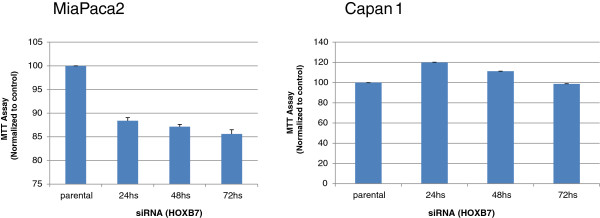
**Colorimetric assay for cell viability (MTT).** The results represent the mean ± standard error of three independent experiments and are presented after normalizing to the respective controls. No significant decreases in cell viability were observed after 24, 48 and 72 hours of HOXB7 siRNA treatment in MIA PaCa-2 or Capan-1 cell lines.

The impact of siRNA transfection on cell viability was investigated after 24, 48, and 72 hours of incubation, using the MTT assay. As shown in Figure 5, no significant differences in absorbance were observed in comparison to the parental cells.

### Flow cytometric analyses of markers of proliferation and cell death

**Figure 6:**
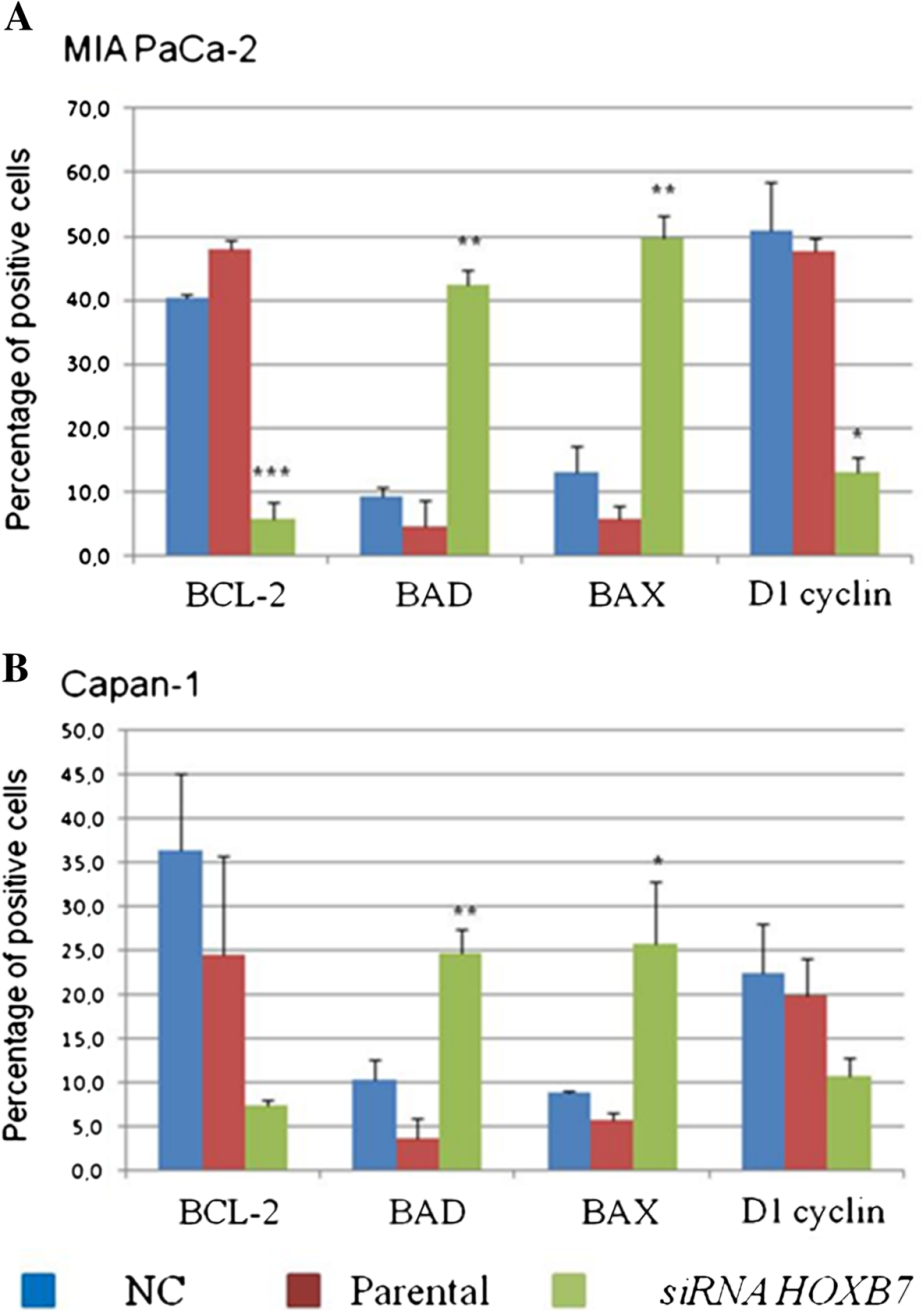
**BCL-2, BAD, BAX and D1 cyclin expression as evaluated by flow cytometry.** Panels **A** and **B** demonstrate MIA PaCa-2 and Capan-1 cells lines, respectively. The experiments were carried out in triplicate and the bars represent mean ± standard deviation. NC- negative control. * p <0.05, ** p <0.01, *** p <0.001.

Modulation of BAX, BAD (pro-apoptotic), BCL-2 (antiapoptotic) and D1 cyclin (marker of cell proliferation) were evaluated after 48 hours of treatment with *HOXB7* siRNA. An increased in the expression of the pro-apoptotic proteins BAX and BAD was observed in both cell lines (p<0.01), while expression of BCL-2 and cyclin D1 were significantly decreased by treatment in MIA PaCa-2 cell line (p<0.01 and p<0.05 respectively) (Figure 6A). In the Capan-1 cell line, there was an increase in the expression of the pro-apoptotic BAX and BAD proteins (p<0.01 and p<0.05, respectively), but BCL-2 and D1 cyclin expression remained unchanged after treatment (Figure 6B).

**Figure 7:**
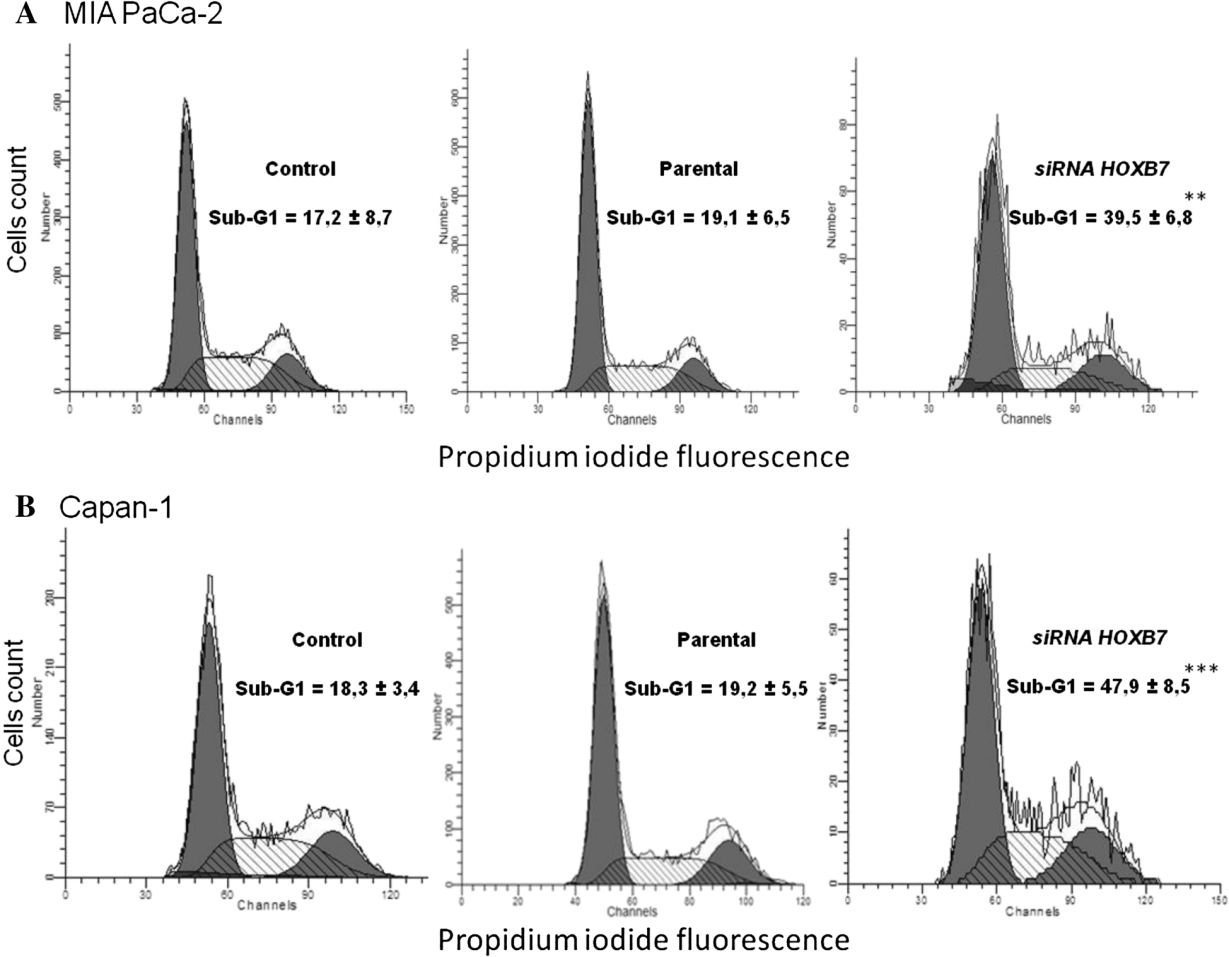
**Distribution of cell cycle phases as evaluated by flow cytometry.** Panels **A** and **B** represent MIA PaCa-2 and Capan-1 lines, respectively. **p<0.01; ***p<0.001.

Cell cycle distribution was assessed after staining fixed cells with PI and thereby cells in different phases of cell cycle were discriminated: G1, S, G2/M; siRNA-transfected cell lines vs. scrambled control and parental demonstrated an accumulation of cells in sub-G1 phase in MIA PaCa-2 (Figure 7A) and Capan-1 (Figure 7B).

**Figure 8:**
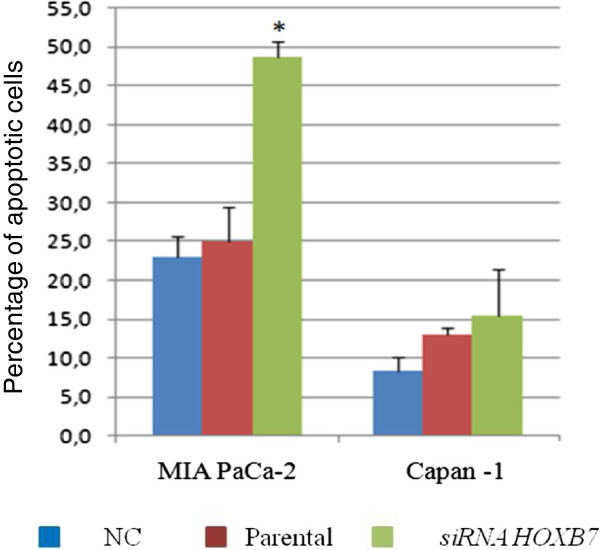
**Percentage of apoptotic cells as evaluated by flow cytometry after treatment with siRNA against to *HOXB7*.** The bars represent mean ± standard deviation. NC- negative control. * p <0.05.

The process of apoptosis was assessed 48 hours after inhibition of *HOXB7* mRNA in the two cell lines studied; only MIA PaCa-2 cell line demonstrated an increase in the percentage of apoptotic cells after treatment (Figure 8).

### Effects of HOXB7 silencing in gene expression profile of PDAC cell lines

In MIA PaCa-2, 679 genes were identified as downregulated after *HOXB7* silencing in comparison to parental cells. The genes were grouped into different categories according to the biological process (Table 1). On the order hand, 12 genes were downregulated and 96 were upregulated in treated Capan-1 cell line compared to parental cells, which were not grouped due to the established statistical cut.

**Table 1:** Biological processes associated with HOXB7 transcript inhibition in MIA PaCa-2 cell lineage

**Biological process**	
Cellular macromolecular complex assembly	C=336;O=32;E=13.19;R=2.43;rawP=3.34e-06;adjP=0.0012
Macromolecular complex subunit organization	C=741;O=55;E=29.09;R=1.89;rawP=3.63e-06;adjP=0.0012
Macromolecular complex assembly	C=672;O=51;E=26.38;R=1.93;rawP=4.49e-06;adjP=0.0012
Organelle organization	C=1339;O=87;E=52.57;R=1.66;rawP=1.38e-06;adjP=0.0012
Cellular macromolecular complex subunit organization	C=396;O=36;E=15.55;R=2.32;rawP=2.42e-06;adjP=0.0012
Protein complex assembly	C=520;O=42;E=20.41;R=2.06;rawP=7.28e-06;adjP=0.0014
Protein complex biogenesis	C=520;O=42;E=20.41;R=2.06;rawP=7.28e-06;adjP=0.0014
Cellular protein complex assembly	C=184;O=21;E=7.22;R=2.91;rawP=1.12e-05;adjP=0.0019
Proteasomal ubiquitin-dependent protein catabolic process	C=107;O=15;E=4.20;R=3.57;rawP=1.79e-05;adjP=0.0022
Proteasomal protein catabolic process	C=107;O=15;E=4.20;R=3.57;rawP=1.79e-05;adjP=0.0022
Interspecies interaction between organisms	C=280;O=27;E=10.99;R=2.46;rawP=1.56e-05;adjP=0.0022
Cell cycle	C=895;O=60;E=35.14;R=1.71;rawP=2.98e-05;adjP=0.0033
Amine biosynthetic process	C=78;O=12;E=3.06;R=3.92;rawP=4.83e-05;adjP=0.0050
Positive regulation of ubiquitin-protein ligase activity	C=67;O=11;E=2.63;R=4.18;rawP=5.37e-05;adjP=0.0052
Positive regulation of ligase activity	C=70;O=11;E=2.75;R=4.00;rawP=8.13e-05;adjP=0.0073
Chromatin organization	C=364;O=30;E=14.29;R=2.10;rawP=0.0001;adjP=0.0084

C- Reference genes in the category, O- number of genes in the gene set and also in the category, E- expected number in the category, R- ratio of enrichment, rawP- p value from hypergeometric test, adjP- p value adjusted by the multiple test adjustment.

### Downregulation of E2F and RB1 genes in MIA PaCa-2 after HOXB7 silencing

**Figure 9:**
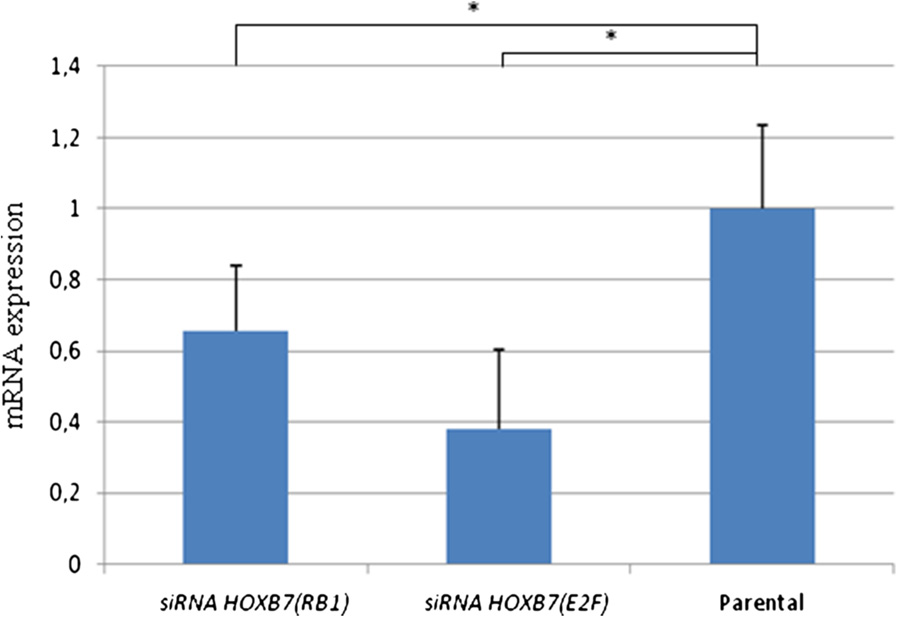
**Validation of microarray assay by qRT-PCR.** RB1 and E2F gene relative expression 48 hours after transfection of HOXB7-specific siRNA. The experiments were carried in triplicate and are represented as mean ± standard deviation *p <0,05.

Among the downregulated genes in MIA PaCa-2 after inhibition of *HOXB7*, we validated the RB1 and E2F transcript expression. As shown in Figure 9, the two downregulated genes were confirmed by quantitative real time PCR.

## Discussion

The main findings of the present study was confirmation of *HOXB7* mRNA overexpression in PDAC as well as the demonstration that its knockdown in two human PDAC cell lines increases expression of the pro-apoptotic proteins BAX and BAD, elicits an accumulation of cells in the sub-G1 phase and modulates cellular gene expression profile.

Nguyen et al. [20] have previously demonstrated overexpression of *HOXB7* mRNA in PDAC, which was positively correlated with lymph node metastasis and considered a predictor of poor prognosis. In that study, knockdown of *HOXB7* by siRNA in the pancreatic cell lines BxPC3, MIA PaCa-2 and PANC1 resulted in decreased invasion but it did not influence cell proliferation or viability as evaluated by the MTT assay [20]. This latter result was also observed in the present study, concomitantly with increased apoptosis as evaluated by flow cytometry in MIA Paca-2 cell line. These apparent discrepant results between the MTT assay and flow cytometry may reflect limitations of the MTT assay, since the metabolic activity measured by this methodology may be changed by different conditions or chemical treatments [30].

Knockdown of *HOXB7* mRNA promoted an increase in the expression of the pro-apoptotic BAD and BAX proteins in both studied cell lines, but the pattern of expression of the anti-apoptotic BCL2 protein differed between them: in MIA PaCa-2, there was a reduction in BCL2 expression, while no significant changes were detected in the Capan-1 cell line. Additionally, downregulation of cyclin D1 also took place only in MIA PaCa-2 cells. The sum of these events may explain the increased apoptosis induced by *HOXB7* siRNA only in MIA Paca-2 cell line.

MIA PaCa-2 and Capan-1 cell lines are derived from pancreatic cancer and we have evaluated both because the first was established from a primary tumor [31] while Capan-1 derived from a hepatic metastasis [32]. They are known to present distinct phenotypic and genotypic characteristics, such as adhesion, invasion, migration, and expression status of commonly altered genes (*KRAS*, p53, p16, and *SMAD4*) [33]. Thus, it is not surprising that these cell lines may exhibit distinct behaviors, as already described in other experimental conditions [33].

According to Hyman et al. gene amplification may be an important mechanism underlying the increased expression of *HOXB7* in breast cancer. However, gene amplification was detected in only 10% of the tested samples [34]. The analysis of *HOXB7* gene copy number in the present study suggests that its increased expression in PDAC does not result from gene amplification, which was identified in only two tumoral samples and in the Capan1 cell line. It is possible that overexpression of *HOXB7* is linked to epigenetic events, which have already been described for other *HOX* family members [35].

Regardless of the mechanism by which *HOXB7* mRNA expression is upregulated in PDAC, we have demonstrated that its knockdown increases apoptosis and also modulates several biological processes only in MIA PaCa-2. Some of the identified biological processes were already described as affected by *HOX* genes in other cell types. For instance we have observed downregulation of genes belonging to proteasomal ubiquitin-dependent catabolic protein process whereas Wang et al. [36] reported that upregulation of *HOXA10* in myeloid cells enhances the protein-dependent ubiquitination of the ubiquitin ligase Triad-1.

We have also shown that suppression of *HOXB7* mainly caused an imbalance in the cell cycle, especially in MIA PaCa-2 cell line, which presented not only downregulation of genes associated with cell cycle in the microarray, but also a reduction of expression of Cyclin D1 in the flow cytometry analysis. This event was also reported by Liao et al. [19], who detected downregulation of cyclin D1 and up-regulation of p27 after *HOXB7* gene silencing with consequent blocking G1-S. Here, we showed *E2F* and retinoblastoma B1 (*RB1*) wich are essential for the G1-S transition. These downregulated transcripts were identified by microarray and confirmed by quantitative real time PCR.

Understanding the molecular abnormalities involved in the pathogenesis of PDAC may reveal new targets for therapy and inhibition of mRNA expression mediated by siRNA can be used to unravel the role of specific genes in the tumorigenic process. In this sense, in the present study, the inhibition of *HOXB7* expression in MIA PaCa-2 and Capan-1 cell lines corroborated the participation of this homeobox gene in the development of PDAC, reinforcing the need for further investigation.

Although the chemotherapeutic agent gemcitabine represents the standard for pancreatic cancer treatment, its use is far from ideal, as prolonged exposure leads to drug resistance. This is a major cause of treatment failure for pancreatic adenocarcinoma and novel therapeutic approaches are needed [37,38]. The use of RNA interference as a therapeutic modality has generated great expectations, however, finding a way to efficiently deliver it to cancer cells is challenging. The inhibition of *HOXB7* by RNA interference in PDAC could be a promising target to be used in combination with conventional chemotherapy.

## Conclusions

*HOXB7* is overexpressed in pancreatic adenocarcinomas and in the two studied pancreatic cell lines; the siRNA assay suggests that *HOXB7* is involved in pancreatic cell proliferation and apoptosis. *HOXB7* is another component of the extensive network of molecules involved in the pathobiology of pancreatic cancer and might constitute a promising target for future biological therapies.

## Competing interests

The authors declare that they have no competing interests.

## Authors’ contributions

RRG designed the study and wrote the manuscript, TC performed most of the experiments with help from MAHZF, RDP and VdJRdP contributed to western blot and bioinformatics analyses, DMA contributed in flow cytometry analyses, MSK provided the tumoral samples, TB performed surgical procedures, MLCG, HMB and EMN critically revised the manuscript and made many conceptual suggestions. All authors read and approved the final manuscript.

## Pre-publication history

The pre-publication history for this paper can be accessed here:

http://www.biomedcentral.com/1471-2407/13/451/prepub
